# Supplementary material for: PolyMOF nanoparticles constructed from intrinsically microporous polymer ligand towards scalable composite membranes for CO2 separation
Source: Nat Commun. 2023 Dec 14;14:8330. doi: 10.1038/s41467-023-44027-y (PMC10721836; doi:10.1038/s41467-023-44027-y)
Supplement: Supplementary file 1 — Supplementary information [file 41467_2023_44027_MOESM1_ESM.pdf]

Supplementary Information

PolyMOF nanoparticles constructed from intrinsically microporous polymer ligand towards scalable composite membranes for CO_2 separation

Tae Hoon Lee[1,2], Byung Kwan Lee[1], Seung Yeon Yoo[1], Hyunhee Lee[2], Wan-Ni Wu[2], Zachary P. Smith[2], and Ho Bum Park[1,*]

[1]Department of Energy Engineering, Hanyang University, Seoul 04763, Republic of Korea

[2]Department of Chemical Engineering, Massachusetts Institute of Technology, Cambridge, Massachusetts 02139, United States

Corresponding Author

*E-mail: badtzhb@hanyang.ac.kr (H. B. Park)

Supplementary Note 1. Synthesis of cPIM-1

An acid hydrolysis method catalyzed by sulfuric acid was used to prepare carboxylated PIM-1 (cPIM-1) due to its fast reaction rate and high conversion rate, which is illustrated in Supplementary Fig. 1[1]. Fourier-transform infrared (FT-IR) spectra of cPIM-1 exhibited the absence of a –CN group at 2240 cm^{-1}, a broad –OH stretch at ~3400 cm^{-1}, and a strong –C=O peak at 1724 cm^{-1}, which confirm the successful conversion of –CN into carboxylic acid (–COOH) groups (Supplementary Fig. 2). Also, the mass loss between 300 and 500°C detected from thermogravimetric analysis (TGA) for cPIM-1 indicates the decomposition of –COOH groups (Supplementary Fig. 3). Due to the strong intermolecular interactions between –COOH groups in cPIM-1 chains, x-ray diffraction (XRD) patterns confirmed that the average pore sizes (i.e., *d*-spacings) of PIM-1 were reduced for cPIM-1 (Supplementary Fig. 4). cPIM-1 showed a lower Brunauer–Emmett–Teller (BET) surface area of 591 m^2 g^{-1} compared to that of PIM-1 (861 m^2 g^{-1}) evaluated by N_2 sorption isotherms at 77 K (Supplementary Fig. 5).

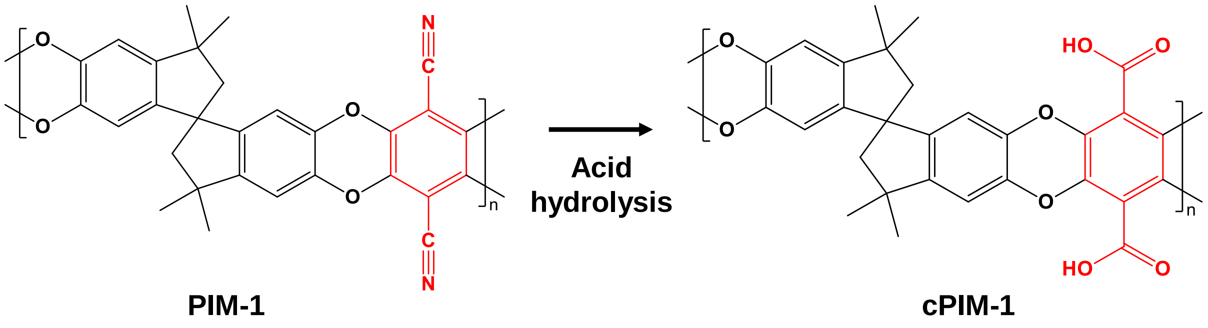

Supplementary Fig. 1. Chemical structures of PIM-1 and carboxylated PIM-1 (cPIM-1).

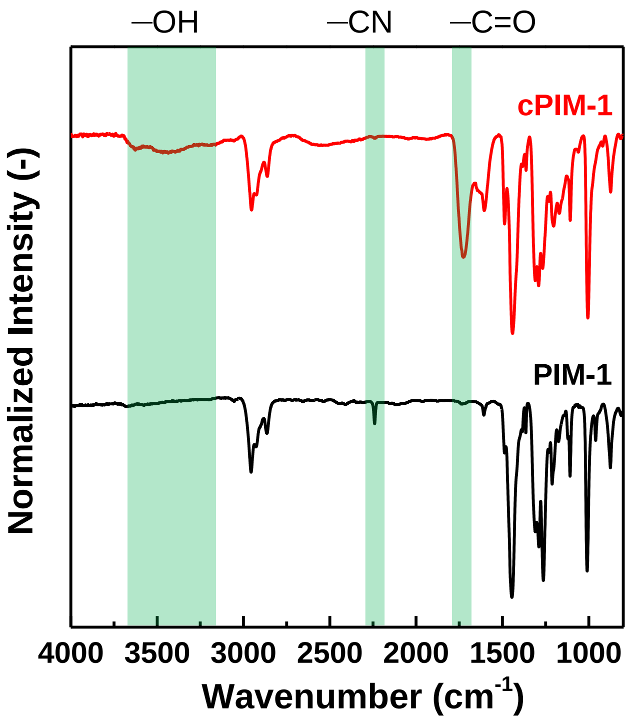

Supplementary Fig. 2. FT-IR spectra of PIM-1 and cPIM-1. cPIM-1 exhibits the absence of a –CN group at 2240 cm^{-1}, a broad –OH stretch at ~3400 cm^{-1}, and a strong –C=O peak at 1724 cm^{-1}.

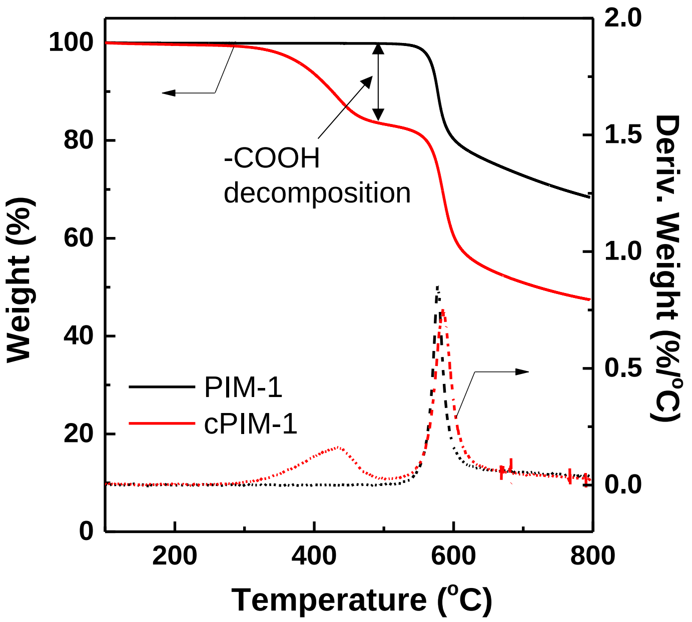

Supplementary Fig. 3. TGA curves of PIM-1 and cPIM-1 measured at 10°C/min ramp rate under N_2 purge.

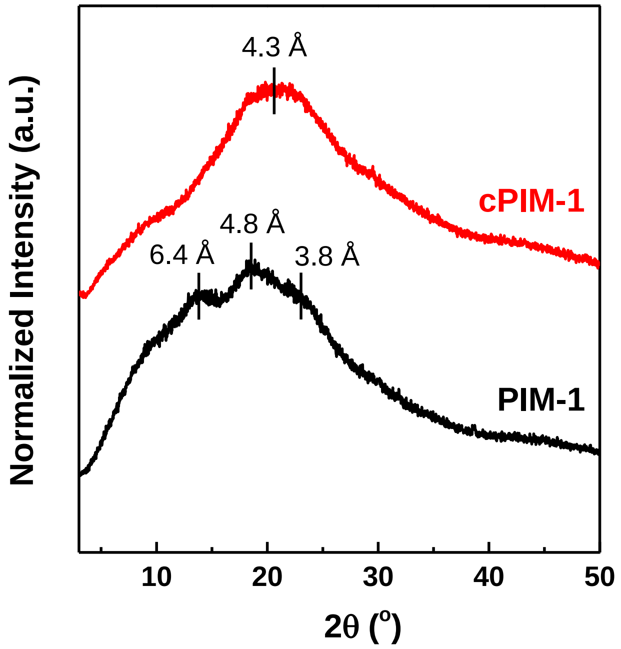

Supplementary Fig. 4. XRD patterns of PIM-1 and cPIM-1.

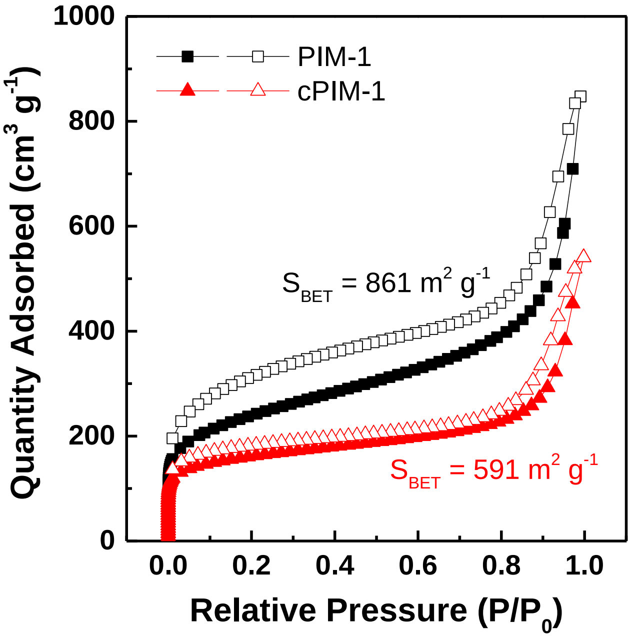

Supplementary Fig. 5. N_2 sorption isotherms at 77 K and BET surface area (S_{BET}) of PIM-1 and cPIM-1.

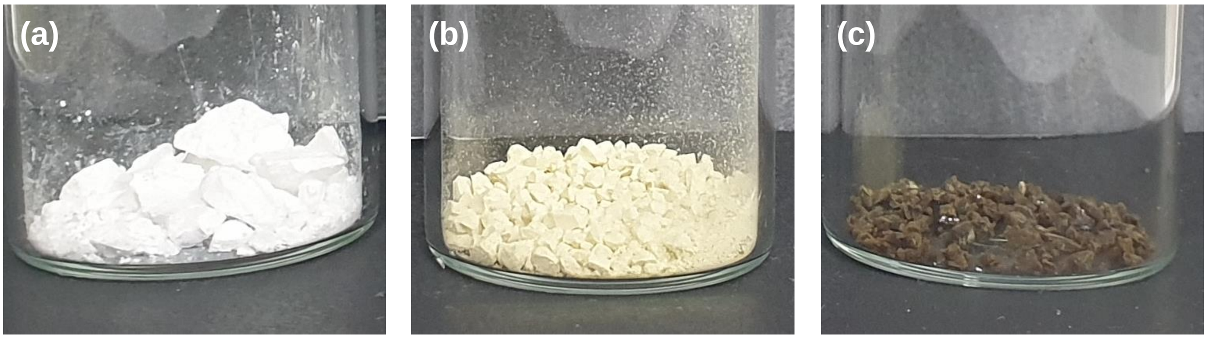

Supplementary Fig. 6. Photo images of (a) UiO-66, (b) polyUiO-66(4:1), and (c) polyUiO-66(0:1) powder.

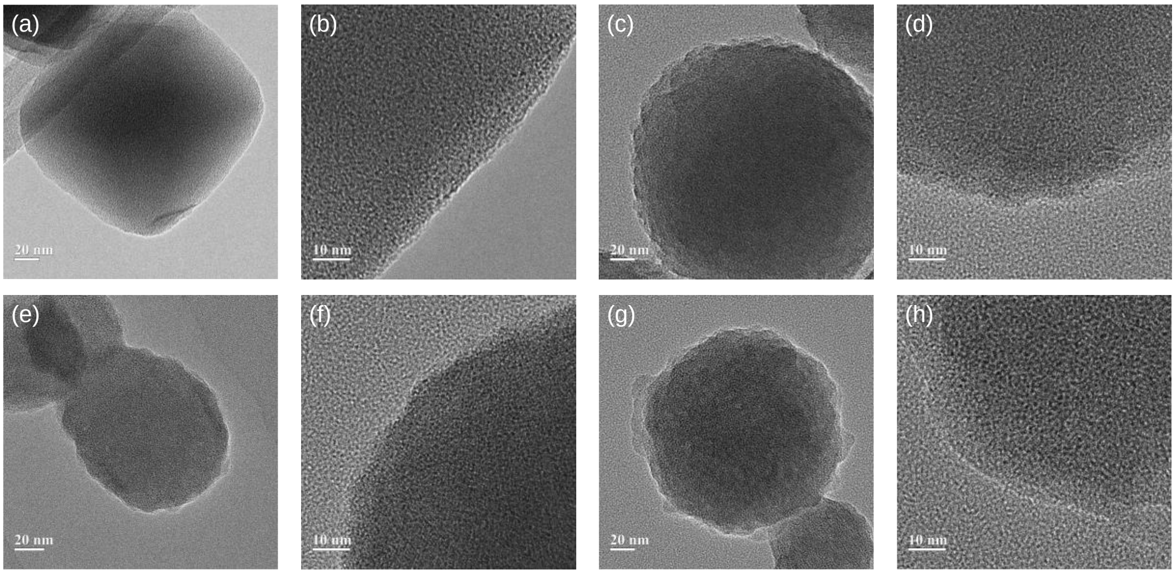

Supplementary Fig. 7. High-resolution TEM images of (a-b) UiO-66, (c-d) polyUiO-66(8:1), (e-f) polyUiO-66(4:1), and (g-h) polyUiO-66(2:1).

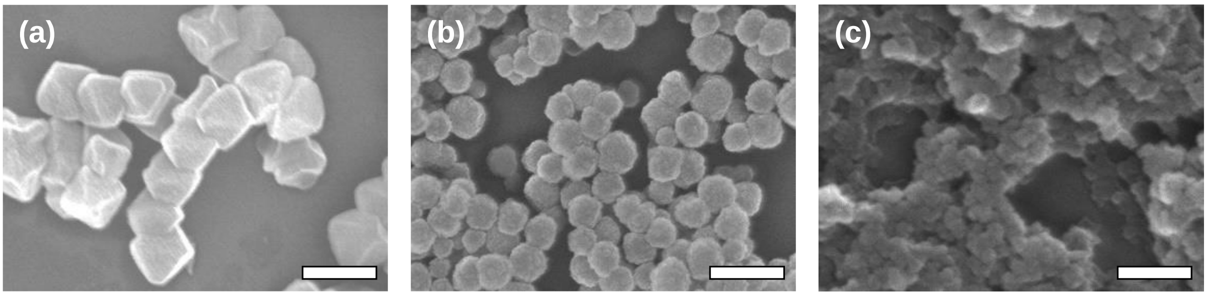

Supplementary Fig. 8. SEM images of (a) UiO-66, (b) polyUiO-66(4:1), and (c) polyUiO-66(0:1) powder (scale bar = 200 nm).

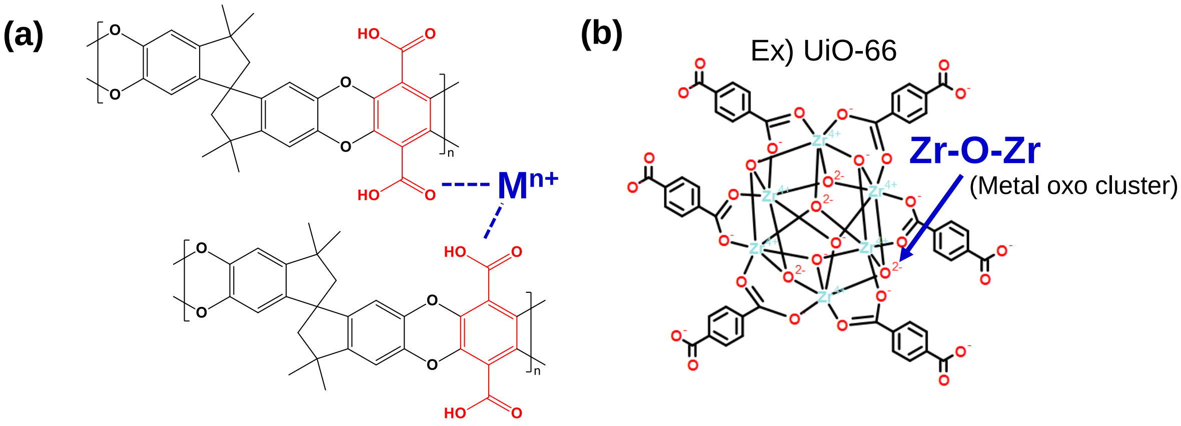

Supplementary Fig. 9. Schematic illustration of possible interactions between metal ions and cPIM-1 ligand: (a) ionic crosslinking and (b) coordination with metal oxo cluster.

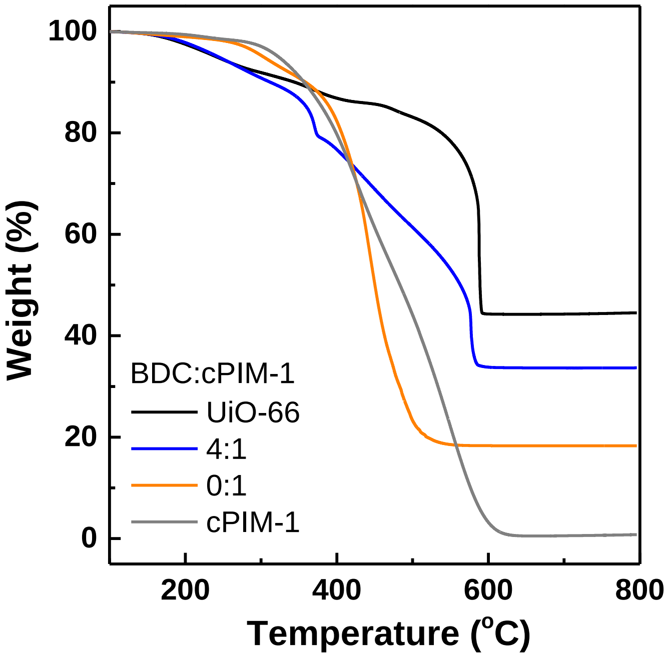

Supplementary Fig. 10. TGA curves of cPIM-1, UiO-66, polyUiO-66(4:1), and polyUiO-66(0:1) measured at 10°C/min ramp rate under air purge.

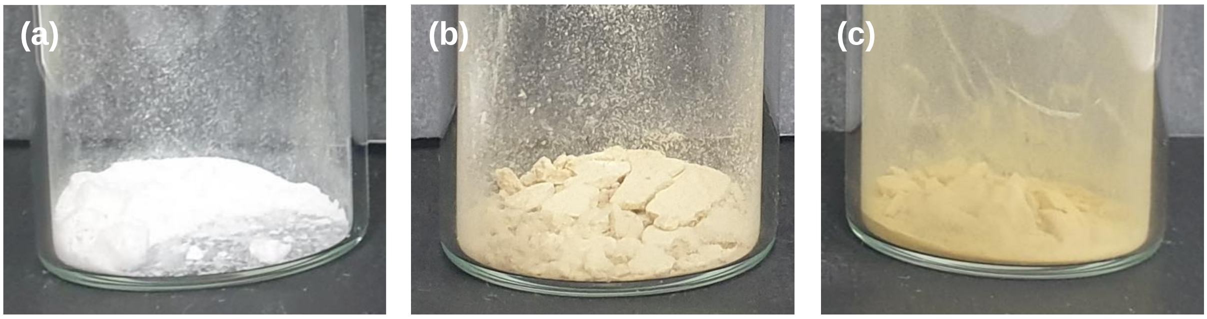

Supplementary Fig. 11. Photo images of (a) MOF-5, (b) polyMOF-5(4:1), and (c) polyMOF-5(0:1) powder.

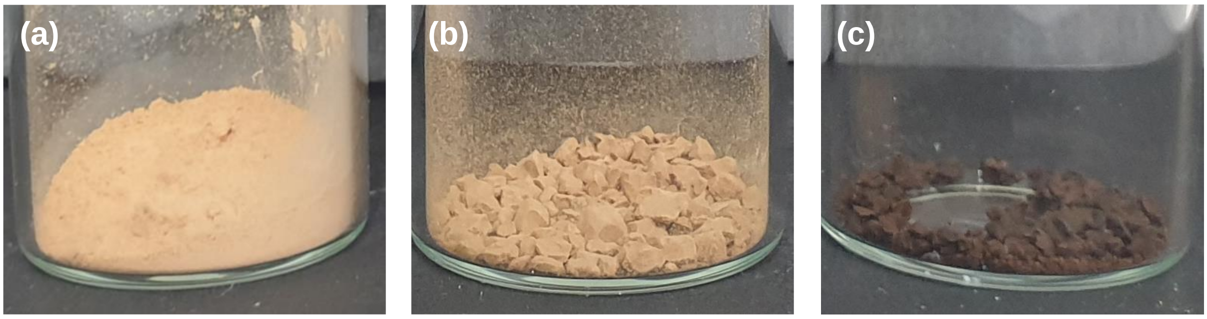

Supplementary Fig. 12. Photo images of (a) MIL-101, (b) polyMIL-101(4:1), and (c) polyMIL-101(0:1) powder.

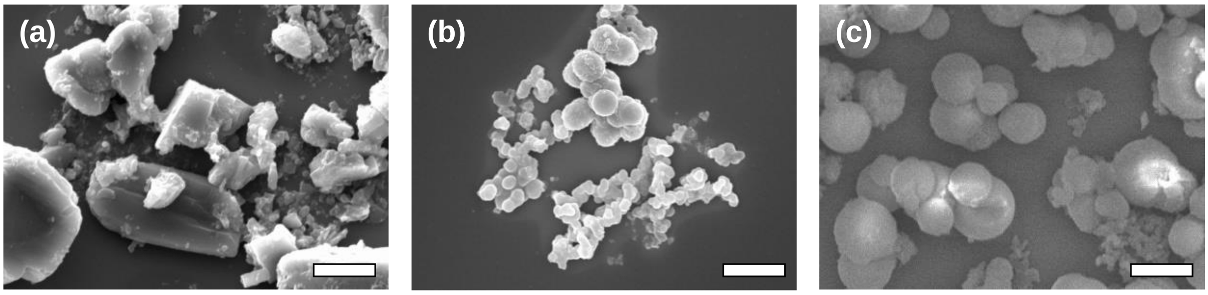

Supplementary Fig. 13. SEM images of (a) MOF-5, (b) polyMOF-5(4:1), and (c) polyMOF-5(0:1) powder (scale bar = 5 µm).

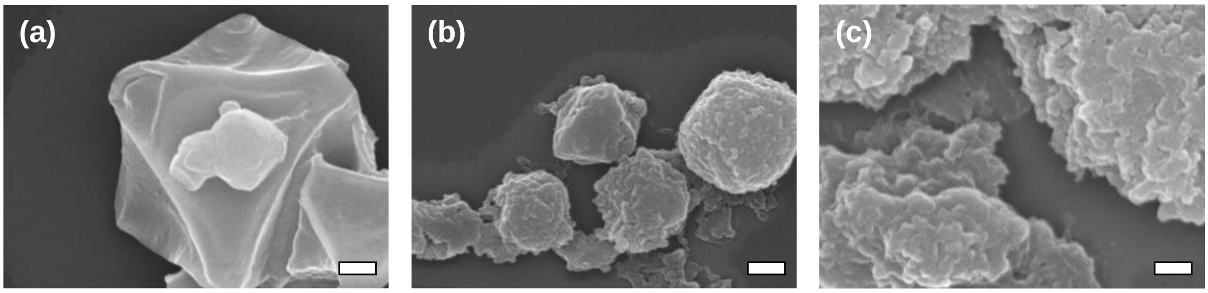

Supplementary Fig. 14. SEM images of (a) MIL-101, (b) polyMIL-101(4:1), and (c) polyMIL-101(0:1) powder (scale bar = 200 nm).

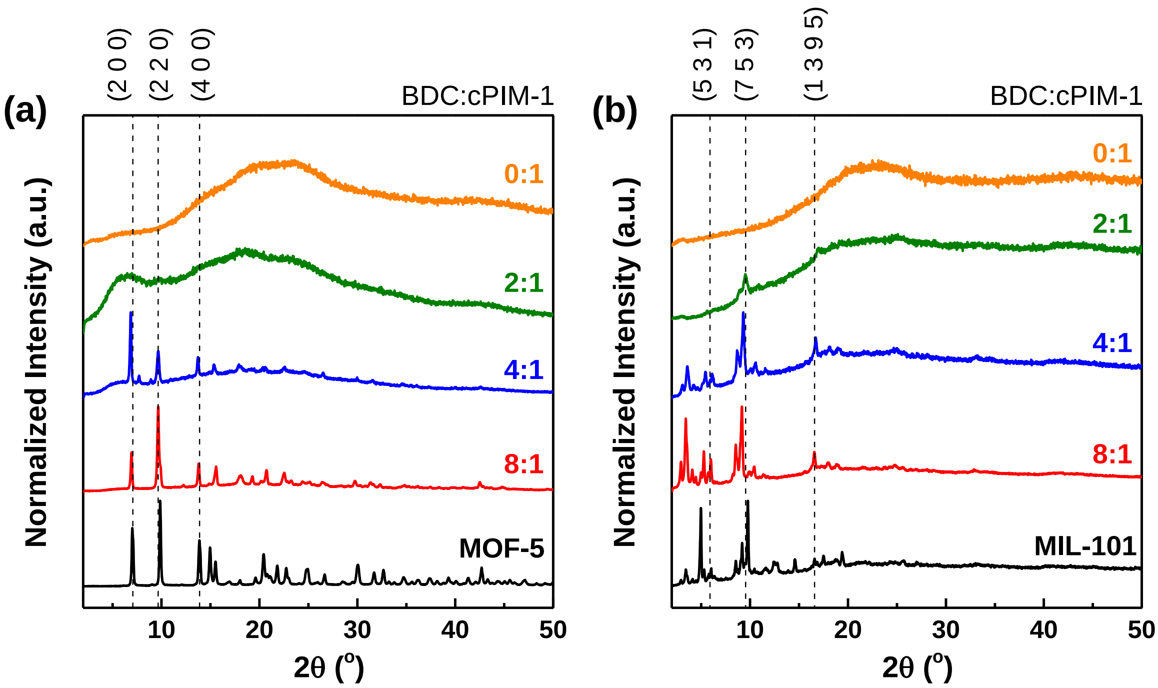

Supplementary Fig. 15. Powder XRD patterns of (a) polyMOF-5[2] and (b) polyMIL-101[3] samples depending on BDC:cPIM-1 ratio used for their synthesis.

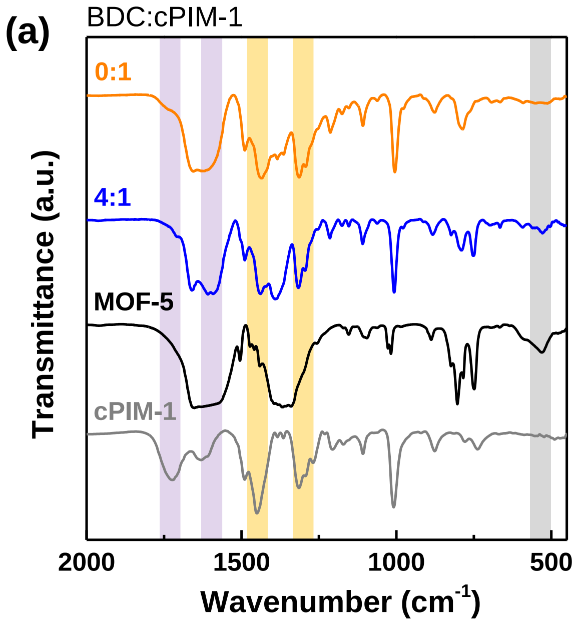

Supplementary Fig. 16. FT-IR spectra of (a) polyMOF-5 and (b) polyMIL-101 samples depending on BDC:cPIM-1 ratio used for their synthesis. The yellow highlighted peaks at ~1450 and ~1310 cm^{-1} correspond to the methylene (CH_2) stretching/bending vibration modes and the C−O stretching mode, respectively. The purple highlighted peaks indicate that the carboxylate bands at 1724 cm^{-1} for cPIM-1 were significantly red-shifted. The gray highlights represent the presence of metal oxo clusters (Zn_4O for MOF-5[4] and Fe_3O for MIL-101[3], respectively).

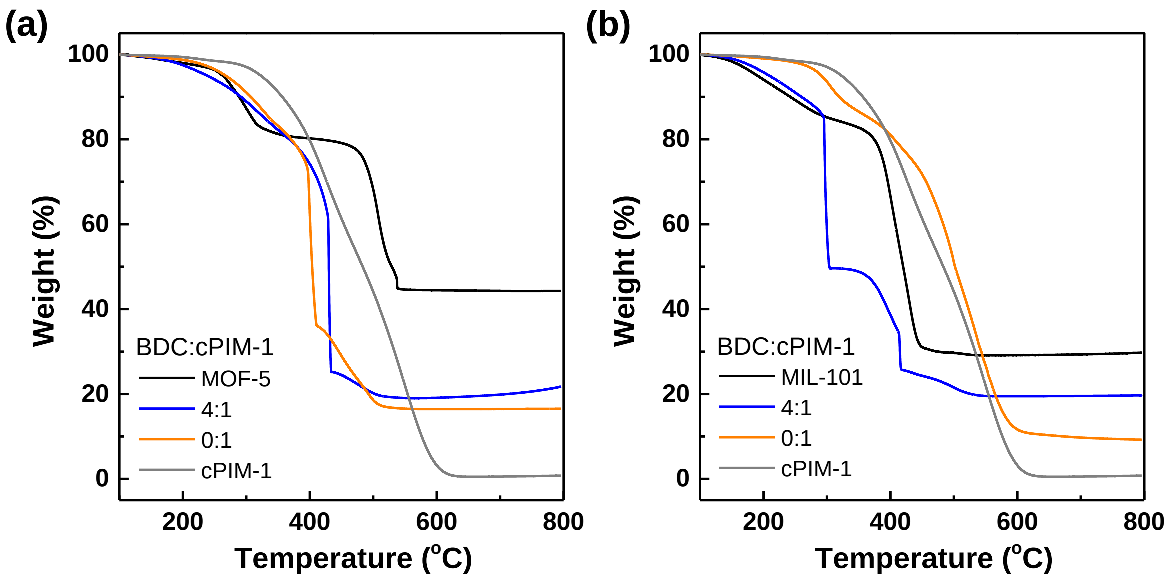

Supplementary Fig. 17. TGA curves of (a) polyMOF-5 and (b) polyMIL-101 samples for various BDC:cPIM-1 ratios used for their synthesis. The weight losses were measured at 10 °C/min ramp rate under flowing air.

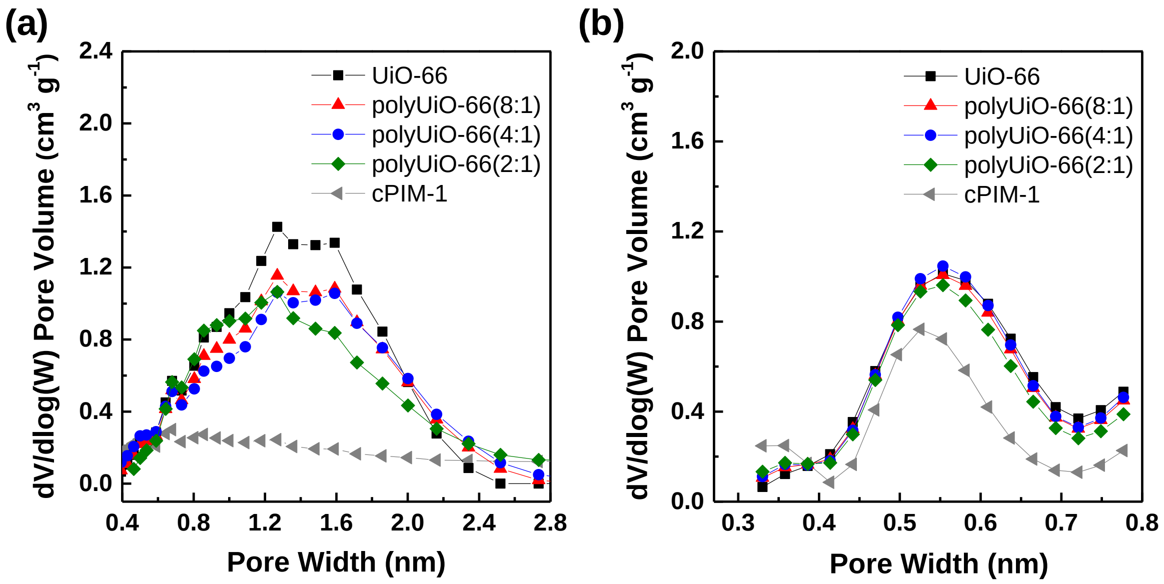

Supplementary Fig. 18. NLDFT pore size distributions of polyUiO-66 nanoparticles calculated from (a) the N_2 adsorption isotherm at 77 K and (b) the CO_2 adsorption isotherm at 273 K. polyUiO-66(0:1) was excluded due to its essentially nonporous nature.

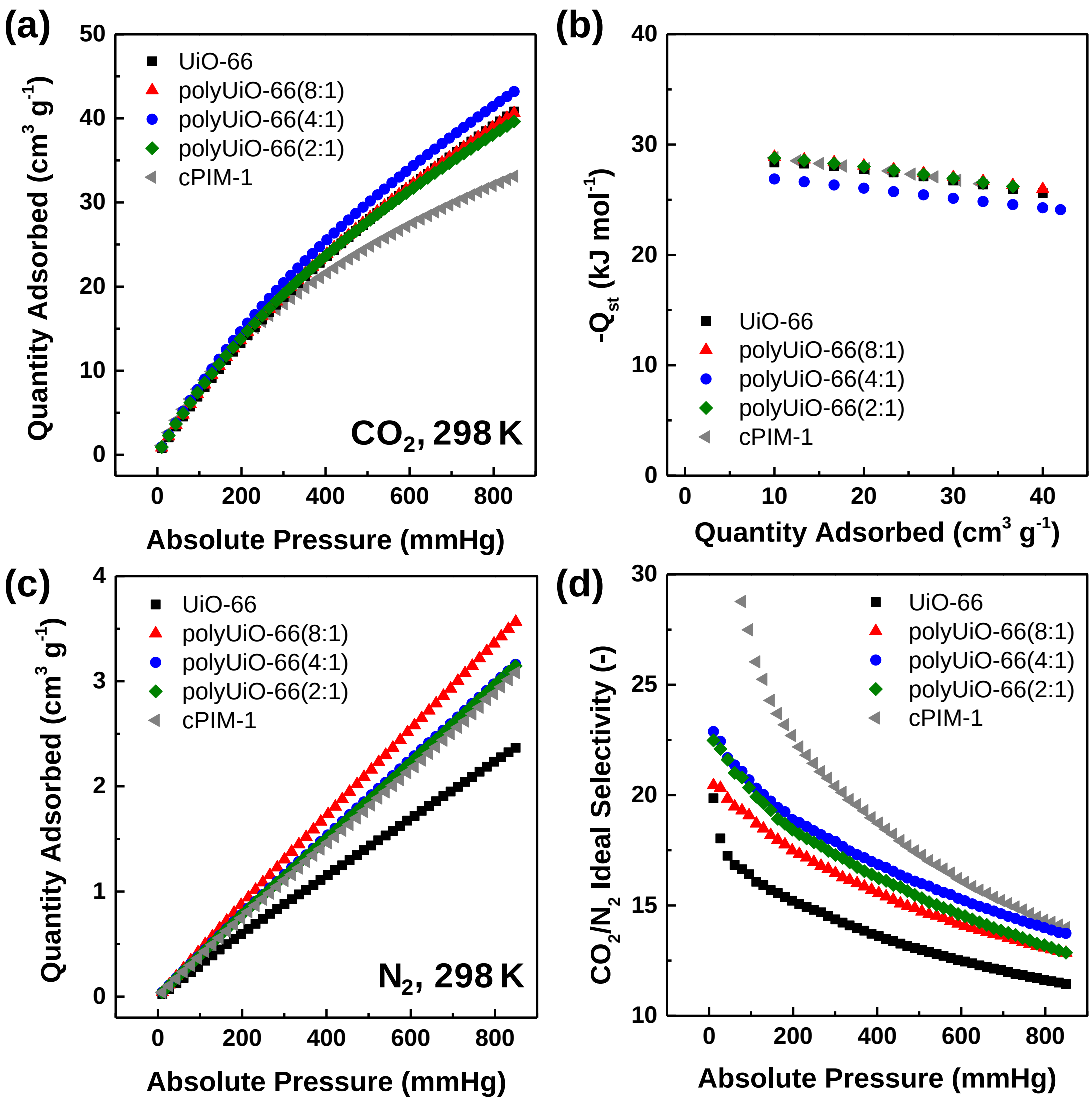

Supplementary Fig. 19. (a) CO_2 adsorption isotherms at 298 K, (b) isosteric heats for CO_2 adsorption, (c) N_2 adsorption isotherms at 298 K, and (d) single gas ideal selectivity calculated from (a) and (c) of polyUiO-66 samples. The single gas ideal selectivity (α) in (d) was calculated as $\alpha = N_{CO2}/N_{N2}$ where N_{CO2} and N_{N2} represent the adsorption amount of CO_2 and N_2 at a certain equilibrium pressure at 298 K[5].

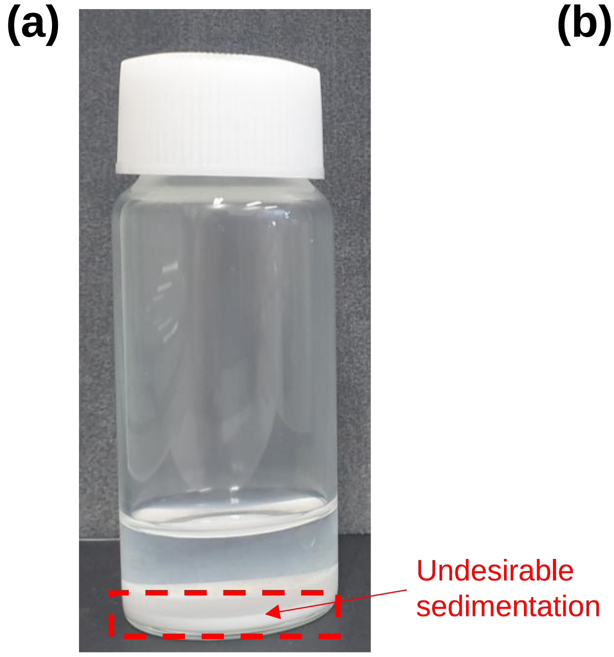

Supplementary Fig. 20. Photo images of the high-concentration (8 mg ml^{-1} in THF) colloidal stability of (a) UiO-66 and (b) polyUiO-66(4:1) nanoparticles 24 h after the as-dispersed state.

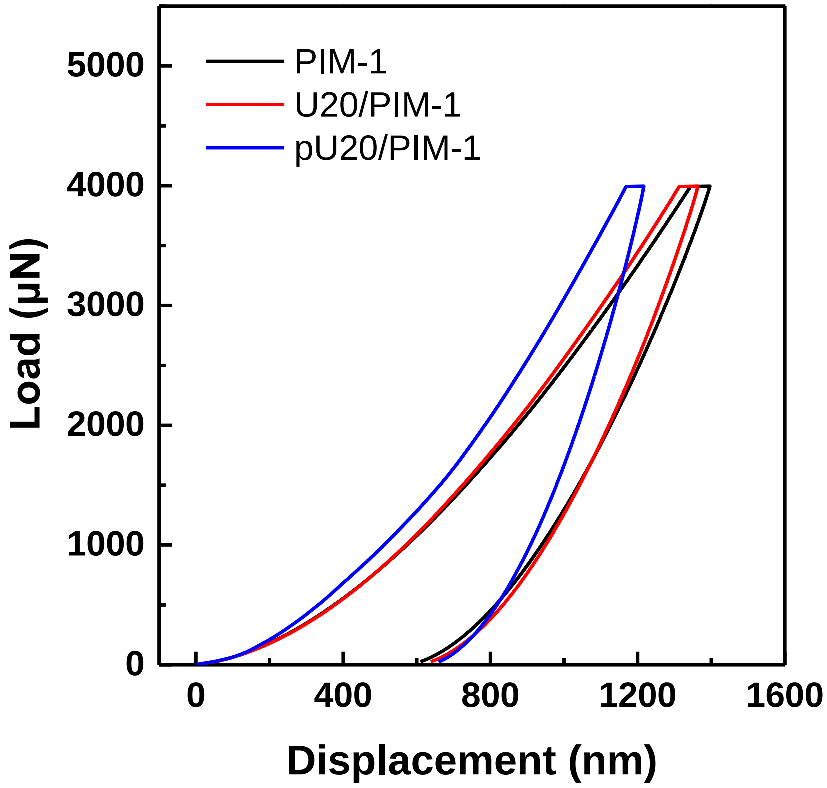

Supplementary Fig. 21. Load-displacement curves of PIM-1 and MMMs from nanoindentations tests.

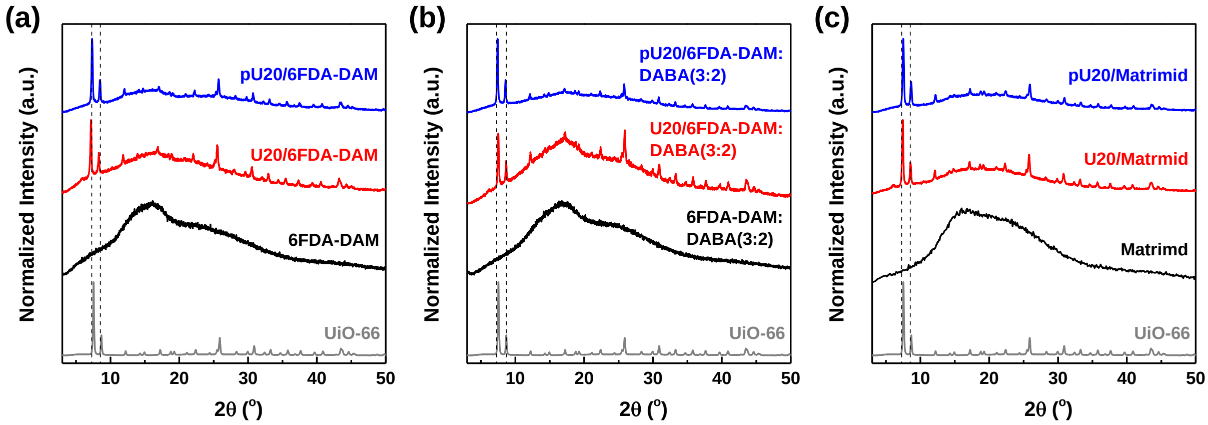

Supplementary Fig. 22. XRD spectra of (a) 6FDA-DAM, (b) 6FDA-DAM:DABA(3:2), and (c) Matrimid MMMs containing 20 wt.% of UiO-66 (U20) or polyUiO-66(4:1) nanoparticles, respectively.

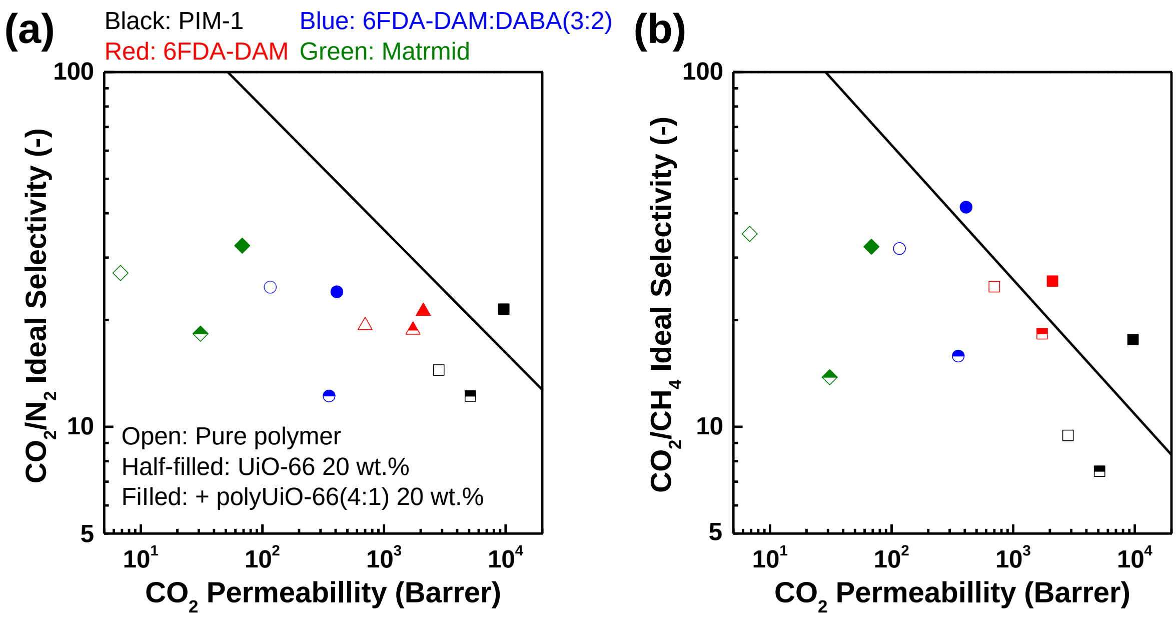

Supplementary Fig. 23. Pure-gas separation performances of pure polymers and MMMs plotted with 2008 Robeson upper bound for (a) CO_2/N_2 and (b) CO_2/CH_4 separation.

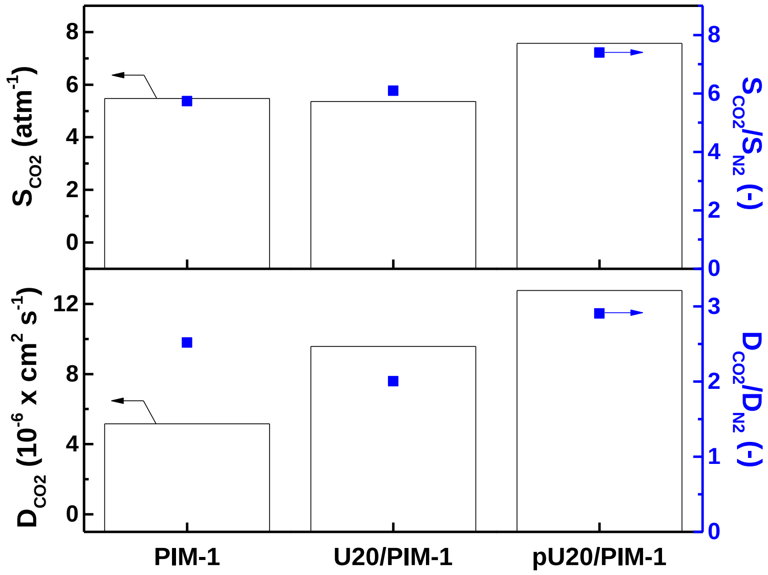

Supplementary Fig. 24. CO_2/N_2 diffusivity selectivity (D_{CO2}/D_{N2}) and solubility selectivity (S_{CO2}/S_{N2}) of PIM-1, U20/PIM-1, and pU20/PIM-1.

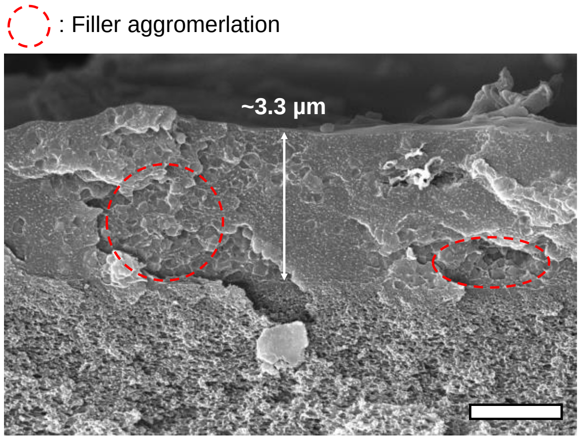

Supplementary Fig. 25. Cross-sectional SEM image of U20/PIM-1 TFN membranes (3×3 cm^2) (scale bar = 2 μm).

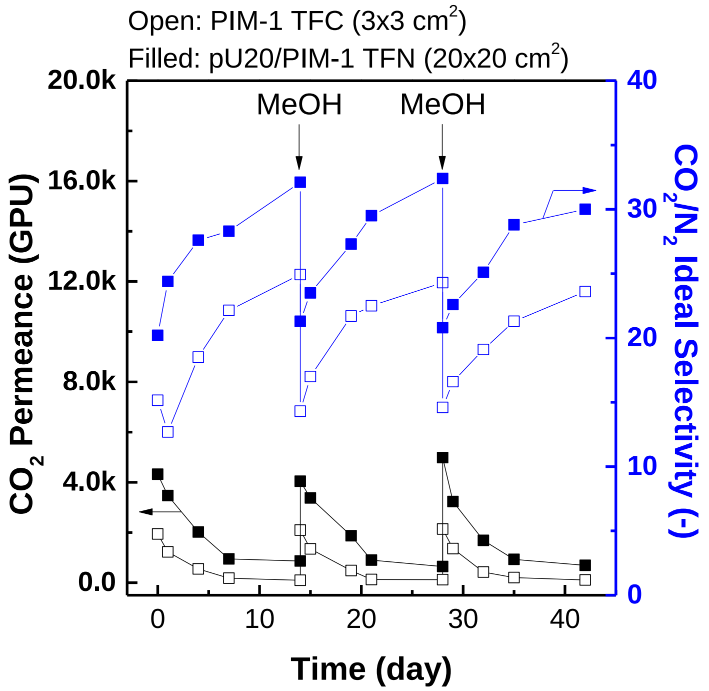

Supplementary Fig. 26. Long-term CO_2/N_2 separation performances of PIM-1 TFC and pU20/PIM-1 TFN membranes with methanol-assisted rejuvenation of the 14-day-aged membranes. For rejuvenation, the aged membranes were soaked in methanol for 24 h and dried in a vacuum oven at room temperature for 24 h.

Supplementary Table 1. Molar ratios of the various reagents used in the MOF and polyMOF syntheses.

Parental MOF	Sample (BDC:cPIM-1)	Metal source (mmol)	Organic (or polymer) ligand (mmol)		Solvent
			BDC	cPIM-1[a]	DMF
UiO-66[b]	1:0	$ZrCl_4$: 0.35	0.35	0	4 mL + Formic acid 0.39 mL
	8:1		0.311	0.039	
	4:1		0.28	0.07	
	2:1		0.233	0.117	
	0:1		0	0.35	
MOF-5[c]	1:0	$Zn(NO_3)_2{\cdot}6H_2O$: 1.20	0.20	0	5 mL
	8:1		0.178	0.022	
	4:1		0.16	0.04	
	2:1		0.133	0.067	
	0:1		0	0.20	
MIL-101(Fe)[d]	1:0	$FeCl_3{\cdot}6H_2O$: 0.735	0.372	0	4.5 mL
	8:1		0.331	0.041	
	4:1		0.298	0.075	
	2:1		0.248	0.124	
	0:1		0	0.372	

a: molecular weight of repeating unit = 498 g/mol
b: reaction temperature: 120°C
c: reaction temperature: 100°C
d: reaction temperature: 110°C

Supplementary Table 2. Concentration of cPIM-1 in polyUiO-66(4:1) and polyUiO-66(0:1) calculated from TGA results.

Sample	Residual mass (wt.%) at 800 °C	Content of cPIM-1 (wt.%)
UiO-66	44.5	0
cPIM-1	0.73	100
polyUiO-66(4:1)	33.7	24.5
polyUiO-66(0:1)	18.3	59.9

Supplementary Table 3. Concentration of cPIM-1 in polyMOF-5(4:1) and polyMOF-5(0:1) calculated from TGA results.

Sample	Residual mass (wt.%) at 800 °C	Content of cPIM-1 (wt.%)
MOF-5	44.3	0
cPIM-1	0.73	100
polyMOF-5(4:1)	21.8	51.6
polyMOF-5(0:1)	16.5	63.8

Supplementary Table 4. Concentration of cPIM-1 in polyMIL-101(4:1) and polyMIL-101(0:1) calculated from TGA results.

Sample	Residual mass (wt.%) at 800°C	Content of cPIM-1 (wt.%)
MIL-101	29.7	0
cPIM-1	0.73	100
polyMIL-101(4:1)	19.7	34.5
polyMIL-101(0:1)	9.3	70.4

Supplementary Table 5. Comparison of CO_2 adsorption at 298 K for various relevant materials.

Adsorbent	BET surface area (m^2 g^{-1})[a]	CO_2 uptake (mmol g^{-1})[b]	CO_2/N_2 Ideal selectivity (-)[c]	$-Q_{st}$ of CO_2 (kJ mol^{-1})[d]	Ref.
UiO-66	1492	1.69	11.8	28.4	This work
polyUiO-66(4:1)	1342	1.79	14.2	26.9	This work
UiO-66(Hf)	940	1.50	11.5	22.8	[6]
UiO-66(Hf)-NH_2	1067	2.80	11.2	25.6	[6]
UiO-66(Hf)-$(OH)_2$	922	4.06	13.1	28.4	[6]
UiO-66(Hf)-$(COOH)_2$	378	1.20	9.2	28.2	[6]
UiO-66(Hf)-$(F)_4$	329	0.82	8.2	23.4	[6]
UiO-66	1730	2.20	12.9	23.3	[7]
UiO-66-$(COOLi)_2$-EX	870	1.65	13.8	26.3	[7]
UiO-66-$(COONa)_2$-EX	869	1.98	11.3	27.7	[7]
UiO-66-$(COOK)_2$-EX	554	1.33	12.1	24.0	[7]
UiO-66-$(COOLi)_4$-EX	970	2.34	11.1	27.5	[7]
UiO-66-$(COONa)_4$-EX	639	1.20	10.0	23.0	[7]
UiO-66-$(COOK)_4$-EX	204	0.61	10.2	24.3	[7]
UiO-66	1120	1.79	11.2	~25	[8]
UiO-66-NH_2	1187	2.98	13.5	~28	[8]
UiO-66-1,4-Naph	766	1.54	12.8	~26	[8]
UiO-66-2,5-$(OMe)_2$	899	2.63	13.2	~32	[8]

a: Measured from N_2 adsorption isotherms at 77 K.
b: Measured from CO_2 adsorption isotherms at 298 K and 1 bar.
c: The single gas ideal selectivity (α) in (c) was calculated as $\alpha = N_{CO2}/N_{N2}$ where N_{CO2} and N_{N2} represent the adsorption amount of CO_2 and N_2 at a certain equilibrium pressure at 298 K.[5]
d: The isosteric heat ($-Q_{st}$) of CO_2 adsorption was calculated from the Clausius-Clapeyron equation.[5]

Supplementary references

1. Rodriguez, K. M. et al. Facile and time-efficient carboxylic acid functionalization of PIM-1: Effect on molecular packing and gas separation performance. *Macromolecules* **53**, 6220-6234 (2020).

2. Greer, H. F. Liu, Y. Greenaway, A. Wright, P. A. Zhou, W. Synthesis and formation mechanism of textured MOF-5. *Cryst. Growth Des.* **16**, 2104-2111 (2016).

3. Li, Z. Liu, X. Jin, W. Hu, Q. Zhao, Y. Adsorption behavior of arsenicals on MIL-101(Fe): The role of arsenic chemical structures. *J. Colloid Interface Sci.* **554**, 692-704 (2019).

4. Arjmandi, M. & Pakizeh, M. Mixed matrix membranes incorporated with cubic-MOF-5 for improved polyetherimide gas separation membranes: Theory and experiment. *J. Ind. Eng. Chem.* **20**, 3857-3868 (2014).

5. Han, G. Rodriguez, K. M. Qian, Q. Smith, Z. P. Acid-modulated synthesis of high surface area amine-functionalized MIL-101(Cr) nanoparticles for CO_2 separations. *Ind. Eng. Chem. Res.* **59**, 18139-18150 (2020).

6. Hu, Z. Nalaparaju, A. Peng, Y. Jiang, J. Zhao, D. Modulated hydrothermal synthesis of UiO-66(Hf)-type metal–organic frameworks for optimal carbon dioxide separation. *Inorg. Chem.* **55**, 1134-1141 (2016).

7. Hu, Z. Faucher, S. Zhuo, Y. Sun, Y. Wang, S. Zhao, D. Combination of optimization and metalated-ligand exchange: An effective approach to functionalize UiO-66(Zr) MOFs for CO_2 separation. *Chem. Eur. J.* **21**, 17246-17255 (2015).

8. Cmarik, G. E. Kim, M. Cohen, S. M. Walton, K. S. Tuning the adsorption properties of UiO-66 via ligand functionalization. *Langmuir* **28**, 15606-15613 (2012).
